# Continued Vigilance – Development of an Online Evaluation Tool for Assessing Preparedness of Medical Facilities for Biological Events

**DOI:** 10.3389/fpubh.2014.00035

**Published:** 2014-04-14

**Authors:** Bruria Adini, Luzie Verbeek, Susanna Trapp, Stefan Schilling, Julia Sasse, Kathrin Pientka, Boris Böddinghaus, Helene Schaefer, Jörg Schempf, Reinhard Brodt, Christian Wegner, Boaz Lev, Daniel Laor, Rene Gottschalk, Walter Biederbick

**Affiliations:** ^1^Prepared Research Center, Ben-Gurion University of the Negev, Beer Sheva, Israel; ^2^Ministry of Health, Tel Aviv, Israel; ^3^Robert Koch Institute, Berlin, Germany; ^4^Municipal Hospitals, Mönchengladbach, Germany; ^5^Health Protection Authority, Frankfurt, Germany; ^6^CSO GmbH Software Solutions, Pforzheim, Germany; ^7^University Hospital Goethe-University, Frankfurt, Germany

**Keywords:** standard operating procedures, measurable indicators, biological event, evaluation of emergency preparedness, disaster management

## Abstract

**Objective:** Effective response to biological events necessitates ongoing evaluation of preparedness. This study was a bilateral German–Israeli collaboration aimed at developing an evaluation tool for assessing preparedness of medical facilities for biological events.

**Methods:** Measurable parameters were identified through a literature review for inclusion in the evaluation tool and disseminated to 228 content experts in two modified Delphi cycles. Focus groups were conducted to identify psychosocial needs of the medical teams. Table-top and functional exercises were implemented to review applicability of the tool.

**Results:** One hundred seventeen experts from Germany and Israel participated in the modified Delphi. Out of 188 parameters that were identified, 183 achieved a consensus of >75% of the content experts. Following comments recommended in the Delphi cycles, and feedback from focus groups and hospital exercises, the final tool consisted of 172 parameters. Median level of importance of each parameter was calculated based on ranking recommended in the Delphi process. Computerized web-based software was developed to calculate scores of preparedness for biological events.

**Conclusion:** Ongoing evaluation means, such as the tool developed in the study, can facilitate the need for a valid and reliable mechanism that may be widely adopted and implemented as quality assurance measures. The tool is based on measurable parameters and indicators that can effectively present strengths and weaknesses in managing a response to a public health threat, and accordingly, steps can be implemented to improve readiness. Adoption of such a tool is an important component of assuring public health and effective emergency management.

## Introduction

### Background

Biological agents do not recognize borders (1) and often spread across continents, causing great concern to governments and the public (2). The various outbreaks that occurred in the past decade such as the severe acute respiratory (3), the avian flu (4), the A/H1N1 pandemic (5), as well as the potential threat of bioterror events, emphasize the need for countries to work together to combat public health crises. A recent study conducted in Europe focusing on prevention and control of communicable diseases stressed the need to prioritize awareness of communicable diseases and provide guidance for best practices, in order to facilitate an evidence-based improvement of emergency preparedness (6).

The increased risk of world-wide infectious disease spread has led to the understanding that a regional and even global response is needed (7). As local and national response impacts on the overall emergency response, collaboration between neighboring as well as distant countries is crucial in achieving preparedness.

Inter-country and global collaboration is orchestrated by the World Health Organization (WHO) through the revised International Health Regulations (7, 8). WHO promotes the following main components in planning and managing public health events: ongoing surveillance of mortality and morbidity; early detection of unexpected, potential internationally spreading biological events; and, continuous assessment of emergency preparedness (8).

Medical facilities are vital components in an effective response to biological events (9). Hospitals and emergency medical services consistently coordinate their work and maintain close collaborations, but primary care institutions often do not participate in emergency-preparedness coalitions (10). Studies have presented that, though there is an increase in appropriate emergency operation plans, information-sharing technologies, new equipment, communication and surveillance systems, as well as enhanced preparedness training programs, there is nevertheless low confidence of medical teams in managing specific biological incidents (2, 11).

Ensuring an effective response to a potential public health/biological event necessitates adoption of evaluation mechanisms. The evaluation enables to identify strengths in capacities and competences that should be sustained and weaknesses that must be rectified (12). In addition, evaluation of disaster preparedness is crucial in order to strengthen national healthcare systems and achieve resilience of health institutions (13). Validated preparedness measures should be applied in order to support accountability and improved outcomes in maintaining emergency preparedness and management (14, 15).

Extensive efforts are made to develop State of the Art means for evaluating emergency preparedness of healthcare systems for the various scenarios. Exercises simulating potential scenarios are used to identify public health systems-level challenges (11). Most exercises are time and resource consuming, and therefore are not implemented as frequently as recommended. Other evaluation means are needed to assure creation and sustenance of emergency preparedness for biological events (16).

To date, there is a lack of a widely approved and adopted evaluation mechanism that facilitates a continuous assessment of preparedness for biological events.

The present study was initiated as a bilateral German–Israeli quest to develop an evaluation tool that will enable to assess preparedness of health services and public healthcare professionals for biological events, and promote emergency preparedness and management of public health/biological events in hospitals and primary health clinics.

## Materials and Methods

### Study design

A joint project, aimed to develop an evaluation tool for biological preparedness, was initiated under the framework of the “Research of Civil Security,” supported by the German Federal Ministry of Education and Research and the Israeli Ministry of Health. A joint German–Israeli steering committee was established consisting of 12 members, experts in emergency management, communicable diseases, public health, and/or healthcare management, as well as software development. The committee worked as a team throughout the development process that lasted from April 2010 to September 2013.

A comprehensive literature review of publications from 1995 to 2012 was conducted using PubMed and Google Scholar, based on the following keywords: biological events, communicable diseases, pandemics and epidemics, emergency preparedness, emergency management, emergency response, measuring emergency preparedness, and evaluation tools. An in-depth analysis of each of the identified articles was implemented in order to extract components relevant to biological events. The components were classified to categories relevant to manage biological events, and were then transformed into measurable parameters that can be integrated in an evaluation tool.

### Selection of participants

In the framework of a modified Delphi process (17), the parameters were disseminated to 228 content experts, 188 from Germany and 40 from Israel. The experts were from various fields of public health and emergency management, including healthcare systems’ managers, front line clinicians, medical professionals, first responders, and infectious diseases specialists.

### Interventions

Two modified Delphi cycles were conducted to achieve a consensus of 75% or higher regarding the evaluation parameters. The content experts were requested to mark their agreement or disagreement to include parameters in the tool, and rank each parameter according to its level of importance. Three levels of importance could be defined – very important (level 1), important (level 2), or less important (level 3).

The Delphi process was run electronically on a protected Internet platform that enabled the respondents to mark their opinions directly in the web-based tool (http://www.delphi-study.com). Most experts were requested to comment on the relevance and importance of 30–50 parameters each. Fifteen experts were requested to evaluate and rank all 188 parameters.

Parallel to the modified Delphi cycles, indicators were integrated in the evaluation tool, describing the level of performance of all elements included in each parameter. Each indicator was designed to allow the evaluator to objectively attest to the level of adherence to the parameter, avoiding the need to base the reported finding on a verbal description provided by the evaluated facility’s representative. The indicators were classified to four potential levels: satisfactory (the aspired benchmark); minor revisions needed; major revisions needed; and, not satisfactory (unacceptable level of performance).

In order to ensure simplicity and objectivity in utilizing the tools, two additional elements were integrated in the evaluation tool for each parameter: (1) description of the source of information/methodology of assessment; and, (2) explanation/full description concerning the appropriate implementation of the parameter in the medical facility.

Following the Delphi cycles, a focus group study was conducted between March 2012 and July 2013, to identify the psychosocial needs of the hospital staff in a biological event, in order to integrate them in the evaluation tool. Groups of two to six people consisting of different professionals in the hospital (medical as well as logistic personnel) were presented with a SARS-like scenario. They were asked to discuss the question: “In a situation like this: what does someone in your position need in order to be able to work professionally and feel safe?” A qualitative data collection method was used to maintain openness for unanticipated emerging themes (18).

The focus groups were recorded and transcribed verbatim. Based on the thematic framework approach by Ritchie et al. (19), the data were analyzed and edited. Results from the analysis were added to the evaluation tool.

Following the feedback from the hospitals’ focus groups, the tool was again revised.

Exercises in six hospitals were conducted to review the applicability of the evaluation tool to their needs. The scenario simulated one index patient accompanied by one contact person that presented to the emergency department of the hospital, arriving a few days after their return from a 2-week vacation in Turkey. While the contact was symptom-free, the index patient was bitten by a tick, suffering from flu-like symptoms, gum bleeding, and nausea caused by an unidentified biological agent.

Prior to the exercise, the hospitals’ administrations received a beta-version of the evaluation tool in order to implement a self-evaluation using the core parameters. They were requested to submit the results to the steering committee within a period of 2–7 weeks, along with suggestions for corrections and comments. Following review of the tool, the hospitals participated in a table-top as well as a practical exercise.

The table-top exercise was dedicated to a presentation of the hospital’s plan to respond to the described scenario. In the practical exercise, actors simulated the scenario at the emergency department. The exercise was completed within 2 h, when an ambulance for infectious patients virtually arrived to transfer the index patient to an isolation unit at the University hospital in Frankfurt on the Main, Germany. In agreement with the hospitals, questionnaires were distributed in order to have a standardized evaluation of the exercise. Approximately 10 observers participated in each exercise, consisting mainly of members of the steering committee. Following the exercises, the hospital teams were requested to analyze their performance in order to adjust their institutional plans, review the evaluation tool, and comment on whether modifications were needed.

### Outcomes

Based on the results that were achieved in the modified Delphi cycles, focus groups, table-tops, and exercises, the evaluation tool was revised. Only parameters that reached the minimum level of consensus (≥75%) were integrated in the tool. The overall level of importance of each parameter was calculated based on the median ranking that was achieved in the second Delphi cycle.

A beta-version of a web-based computerized system was developed, aimed at enabling each hospital or community clinic independent use of the evaluation tool. The system was designed for use by both the individual medical facility and/or a governing authority, such as the management of a cooperation of hospitals and clinics or the Israeli Ministry of Health.

### Analysis

In order to generate the weight of each parameter, an algorithm with the following data was used: the importance of the category, the value of the parameter according to the level of importance (very important → value 10, important → 5, less important → 1), and the number of parameters in each category.

**Figure 1:**
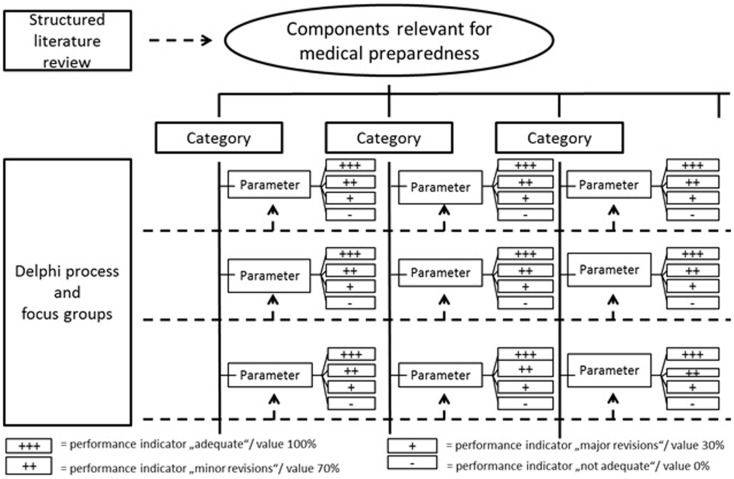
**Methodology for developing the evaluation tool**.

The methodology for developing the evaluation tool is presented in Figure 1.

## Results

One hundred articles were identified as relevant to the scope of the study – emergency management of a biological event. The various subjects discussed in the articles were classified into five categories: policy and planning, medical management, personnel, communication, and infrastructure. The components of emergency management from the five categories were analyzed and then portrayed to define 188 measurable parameters.

### Characteristics of study subjects

The parameters were disseminated to 228 content experts; 188 from Germany and 40 from Israel. Overall response rate for the first Delphi cycle was 51.3% (117 experts). Comparison of replies from Germany and Israel presented response rates of 46.8% (88 experts) and 72.5% (29 respondents), respectively. The desired level of consensus (≥75%) was achieved for most of the parameters; after the first round 176 out of 188 parameters were rated by more than 75% of the content experts for inclusion in the evaluation tool. In addition, 1,031 comments and suggestions were given by the experts, which provided an opportunity to portray the decision-making considerations in the next Delphi cycle.

Despite the high consensus levels achieved in the first cycle, the ratings as well as the comments of the experts were aggregated and disseminated to the respondents for a second modified Delphi cycle. Overall response rate for the second modified Delphi cycle was 55.6% (65 respondents out of 117), based on 45.5% (40 out of 88 experts) from Germany and 86.2% (25 out of 29 experts) from Israel.

### Main results

One hundred eighty-three parameters reached the desired level of consensus (≥75%) in the second Delphi cycle and were then integrated in the evaluation tool. Two hundred nine comments and suggestions were given in the second cycle. The level of consensus regarding the parameters in the two modified Delphi cycles are presented in Table 1.

**Table 1:** **Level of consensus in the two modified Delphi cycles**.

Category	Total number of parameters	Delphi cycle I – % of parameters >75% consensus	Delphi cycle II – % of parameters >75% consensus
Policy and planning	56	91% (51)	100% (56)
Medical management	38	95% (36)	97% (37)
Personnel	46	89% (41)	91% (42)
Communication	29	100% (29)	100% (29)
Infrastructure	19	100% (19)	100% (19)
Total	188 parameters	176 parameters	183 parameters

**Figure 2:**
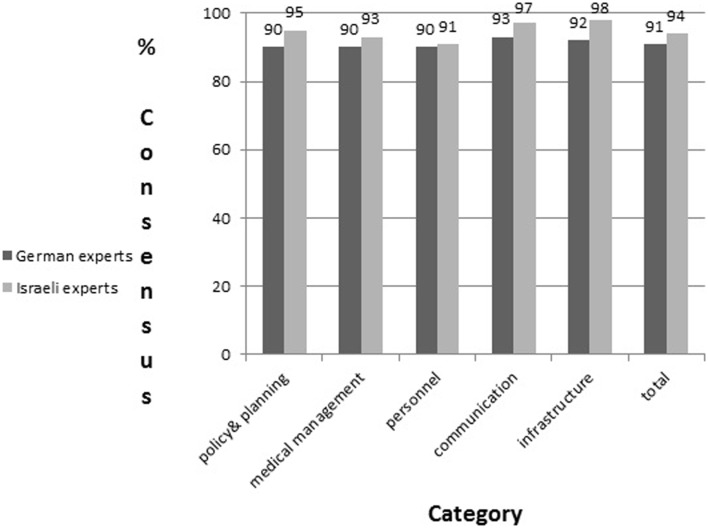
**Comparison of consensus levels (in percentage) among German versus Israeli content experts’ in the modified Delphi cycles**.

Comparison of the views of the German versus Israeli content experts, as expressed in the two modified Delphi cycles, presents a strong similarity concerning elements that should be integrated in the evaluation tool. Overall, 91% of the experts from Germany versus 94% of the respondents from Israel expressed their support in integrating the 183 parameters in the evaluation tool. The level of consensus from both countries concerning each category is presented in Figure 2.

In addition, the parameters were classified based on the characteristics of the hospitals in regard to the competence and responsibility concerning infectious diseases (module). Three modules were defined: (1) core (hospitals without specialization in infectious diseases and/or infectious disease consultants); (2) intermediate (hospitals with isolation facilities able to treat a limited number of patients with highly infectious diseases); (3) advanced (hospitals responsible for management of large numbers of highly infectious disease patients).

In order to rank the relative importance of each of the five categories, a mini-Delphi was conducted among the members of the steering committee (*n* = 9) and the mean was calculated. The relative importance of the categories was determined as follows: medical management – 25.6%; personnel – 25%; policy and planning – 20.6%; infrastructure – 15.6%; and, communication – 13.2%. These data were further used to calculate the overall weight of each parameter in the context of its category by using the following formula: weight = importance of category × value/no. of parameters in category.

One focus group was initiated in Israel, consisting of five members of the Biological National Advisory Committee, amongst them three physicians and one support team member (all males), and one female nurse. Eleven focus groups were conducted in Germany (consisting of staff from six hospitals). The participants included 54 professionals (36 nurses, 9 physicians, and 9 support team members), among them 17 men and 37 women, consisting of two to six participants in each meeting. Emerging themes in the discussions focused mainly on information flow, problems with hierarchy, organization and leadership in case of an event, and personal protective equipment.

One additional element was found by the focus groups in Germany to be lacking in the evaluation tool. After analysis of the data raised in the focus groups, further information could be provided as auxiliary knowledge. Thus, 1 parameter and 40 supplements (recommendations, additional information, or suggestions as to how to solve common problems) were suggested for inclusion. No changes to the evaluation tool were recommended by the Israeli focus group.

Four hospitals in Hesse (HS) and four in Rhineland-Palatinate (RLP) were recruited in Germany for the exercises. Two of the four hospitals in HS belonged to the same agency and in RLP, three of the four were run by the same administration.

The evaluation tool was finalized after being modified according to the comments and suggestions recommended in the Delphi cycles, discussions within the steering committee, input received from the focus groups and feedback from the hospital exercises. The result was deletion of 11 parameters from the evaluation tool, due to a strong similarity to other parameters (7 parameters), current lack of technology (one parameter), or because they fall under the responsibility of the public health services rather than the hospitals or clinics (4 parameters). In addition, one parameter was added from the focus group study, thus the final evaluation tool consisted of a total of 172 parameters.

The median level of importance of each parameter was calculated based on the ranking recommended by the content experts in the Delphi process. As one parameter originated from the focus groups, it was not ranked in the two Delphi cycles. Therefore, the steering committee (*n* = 6) voted for its importance. Twenty-four parameters were ranked as very important, 120 as important, and 28 as less important. Classification of the parameters in each category according to their importance in the finalized evaluation tool is presented in Table 2.

**Table 2:** **Classification of parameters in the final evaluation tool according to level of importance**.

Category	Importance			Total
	Very important	Important	Less important	
Policy and planning	8	37	5	50
Medical management	3	23	10	36
Personnel	4	28	9	41
Communication	6	19*	2	27
Infrastructure	3	13	2	18
Total	24	120	28	172

*Including one parameter as a result of the focus groups.

The final version of the evaluation tool is structured as follows: category, subcategory, module, functionary, area of operation, parameter, level of importance, source of information/evaluation technique, four levels of performance, and additional explanation. The evaluation tool includes 172 parameters, classified to 5 categories and 15 subcategories. An example of the parameters and indicators integrated in the evaluation tool is presented in Table 3.

**Table 3:** **An example of the parameters and indicators integrated in the evaluation tool**.

ID	Category	Parameter	Performance evaluation: satisfactory	Performance evaluation: minor revisions needed	Performance evaluation: major revisions needed	Performance evaluation: not satisfactory	Parameter weight
157	Communication and mental health	Staff should be offered resilience training before, during, and after biological events that specifically addresses the special circumstances of biological incidents (e.g., infection risk)	Resilience training or similar courses are offered at least once a year, are well attended and accepted; all persons interested are given the opportunity to attend	Staff is offered the opportunity to attend resilience training or a similar course once a year; however, interest is low or not everyone interested can manage to attend	The hospital does not proactively arrange for further training/courses in this field	The hospital does not encourage resilience training	2.5
13	Infrastructure	The isolation facility should provide adequate gas exchange installations, such as oxygen supply, according to the number of critical care beds to be provided depending on public health planning	Isolation rooms for at least five critically ill patients exist; each bed place equipped with medical gas outlets	Isolation rooms for at least five critically ill patients do exist, but only two to three bed places are equipped with medical gas outlets	Isolation rooms for at least five critically ill patients do exist, but only one bed place is equipped with medical gas outlets	Isolation rooms for at least five critically ill patients do exist, but no bed place is equipped with medical gas outlets	4.3
272	Medical management	ED personnel should have rapid access to treatment algorithms for patients in a biological event	Accessible within 15 min	Accessible within 30 min	Accessible within 45 min	Not accessible within 45 min	3.5
231	Policy and planning	A prioritized hospital biological preparedness plan should be updated for the last year	Hospitals designated to handle biological incidents update their hospital contingency plans every year	The plan is updated for the past 2 years	The SOP is updated for the past 3 years	Hospitals designated to handle biological incidents do not update their hospital contingency plans	2.1
82	Personnel	The triage staff should know how to separate individuals suffering from the psychological consequences of a bioterrorist attack from individuals suffering from physical disorders	Triage staff knows the case definition in case of a contingency and are able to distinguish it from symptoms/complaints that are indicative of a primarily psychological trauma	Triage staff knows the case definition in case of a contingency but are not able to distinguish it from symptoms/complaints that are indicative of a primarily psychological trauma	Triage staff knows the case definition in case of a contingency but are not familiar with symptoms/complaints that are indicative of a primarily psychological trauma	Triage staff knows neither the case definition in case of a contingency nor are they familiar with symptoms/complaints that are indicative of a primarily psychological trauma	3

A web-based software tool was developed for use during the evaluation process. The tool is structured in three tiers to allow non-complicated utilization by the different users, as well as flexibility in integrating changes. New applications or content can easily be developed, without need for major changes, thus a continuous update and maintenance are assured. ASP-NET technology from Microsoft was used for the web application and the data base system is Microsoft SQL server. Taking into consideration the different clients, the system was localized in three languages – English, German, and Hebrew. The software service enables to record level of performance regarding each parameter and can then produce a report of the level of preparedness of the facility. The report includes scores of the emergency preparedness according to the different categories of emergency response for biological events and the relevant scaling (rank) of each parameter, as well as a list of all elements that were found as needing modifications or performed in an unsatisfactory way.

### Limitations

A few limitations should be noted with respect to the bilateral study. The parameters and indicators were initially developed in English and then translated to German and Hebrew, respectively. It is possible that some of the meanings were distorted in the translation process. In effort to overcome this risk, the joint steering committee reviewed each parameter several times, checking the translation from English to the two respective languages and its re-translation back to English. The Delphi cycles in Israel were conducted in English rather than in Hebrew; this might have impacted on the response rates.

The experts participating in the Delphi cycles were asked about the relevance of parameters but not for completeness of content. It cannot be excluded that certain parameters might have been approved, though the content was not offered by the steering committee. To overcome this possibility, apart from the comprehensive literature review, hospital exercises and focus groups were conducted.

For further evaluation, the data protection laws in Germany limit the ability to conduct comprehensive comparisons of results concerning levels of emergency preparedness of each medical facility.

## Discussion

An outbreak of an infectious disease may cause significant damage to any society, and has the potential to rapidly spread and impact countries worldwide (1, 2). Considering the severe risk, effective preparedness and management of such an event are crucial components of emergency response (7). Assuring an effective response for biological threats necessitates creation and sustenance of a reliable and valid evaluation process that can be implemented continuously (12, 13).

Various tools are utilized globally to evaluate readiness of hospitals and other medical facilities to manage emergency scenarios (11, 16). Nonetheless, these tools are not suitable for biological events (2, 9, 11), due to the unique characteristics of disease outbreaks (incubation periods, gradual development of symptoms, need for surveillance systems to monitor morbidity and mortality, challenge of detection and identification, specific pharmaceuticals, particular protective gear, isolation capabilities, etc.).

The BEPE project initiated jointly by Germany and Israel under the framework of the “Research of Civil Security” was designed to develop such an evaluation tool that will enable to identify strengths and weaknesses in the preparedness system and improve gaps. The tool is targeted to save lives upon an occurrence of a public health threat/biological event. The parameters integrated in the evaluation tool serve as benchmarks for the healthcare facilities that delineate actions that need to be implemented in order to achieve preparedness for biological events (20). The parameters enable both the governing authorities and each facility to independently evaluate the level of readiness.

Development of clear and well-defined indicators is a vital component of any evaluation tool. Indicators are aimed to clearly present the level of performance of each parameter. They provide focus and direction to help identify strengths and gaps in the emergency-preparedness levels and facilitate target quality improvement efforts (11, 21). As recommended in other studies (22), the indicators in the present tool were developed through the adoption of the SMART criteria (specific, measurable, achievable, relevant, and time-bound). Through the modified Delphi process, validity concerning the parameters was achieved as well as promotion of inter-rater reliability thus enabling to draw conclusions regarding the effectiveness of the medical institutions to manage biological events (23). The indicators are classified to four levels, in order to encourage improvement. Considering the complexity, needed resources and efforts that must be invested in each activity designed to increase preparedness, integration of less than four levels of performance may cause inability to witness a positive trend of correcting gaps. Providing the opportunity to display even small advancements empowers the health facilities’ administrations in their efforts to achieve quality improvement.

The high resemblance of the responses of the German versus the Israeli experts in the modified Delphi process supports the perception that preparedness for biological events is similar worldwide (6, 8). Characteristics of Germany and Israel differ in many aspects such as size (populations of 81.5 versus 8 million, respectively); type of government (Israel is characterized by a centralized government while Germany is a federation of states); responsibility of each hospital regarding biological events (in Germany there are three tiers of hospitals classified according to preparedness for a single patient, intermediate or mass casualty events while Israeli hospitals are all required to be ready for mass casualties). The evaluation tool is targeted in Germany for the independent use of each medical facility, while in Israel it is targeted not solely for the facility’s administration but also to the Ministry of Health and other governing authorities. Nevertheless, experts of both countries found the parameters included in the evaluation tool appropriate and relevant for biological preparedness, thus signifying that the developed tool can most probably be adopted as a generic tool. Adoption of such an ongoing means of quality improvement mechanism is crucial to all societies (24) and may contribute to creating and maintaining capacities to respond to potential biological events. Implementation of such mechanisms in multiple systems may enable to identify global trends and thus increase public health and safety.

In summary, infectious and communicable diseases may impact on the public health of the world’s population; therefore it is of global interest to maintain a continuous high level of alert and emergency preparedness to manage such events. An ongoing evaluation means, such as the tool that was developed in the present study, may facilitate the need for a valid and reliable mechanism that can be widely adopted and implemented as part of quality assurance measures. The developed tool is based on measurable parameters and indicators that can effectively present strengths and weaknesses in managing a response to a public health threat, and accordingly, steps can be implemented to improve the readiness. Adoption of such a tool is an important component of attaining and assuring public health and effective emergency management.

## Author Note

Link to the computerized tool: http://www.be-prep.com/us

## Conflict of Interest Statement

The authors declare that the research was conducted in the absence of any commercial or financial relationships that could be construed as a potential conflict of interest.
